# Methadone-mediated sensitization of glioblastoma cells is drug and cell line dependent

**DOI:** 10.1007/s00432-020-03485-3

**Published:** 2020-12-14

**Authors:** Bodo Haas, Janine Ciftcioglu, Sanja Jermar, Sandra Weickhardt, Niels Eckstein, Bernd Kaina

**Affiliations:** 1 Federal Institute for Drugs and Medical Devices, Kurt-Georg-Kiesinger-Allee 3, 53175 Bonn, Germany; 2 Faculty of Applied Natural Sciences, Cologne University of Applied Sciences, Kaiser-Wilhelm-Allee, 51368 Leverkusen, Germany; 3 Applied Pharmacy, University of Applied Sciences Kaiserslautern, Campus Pirmasens, Carl-Schurz-Str. 10-16, 66953 Pirmasens, Germany; 4 Institute of Toxicology, University Medical Center, Obere Zahlbacher Strasse 67, 55131 Mainz, Germany

**Keywords:** Glioblastoma, Methadone, Temozolomide, Doxorubicin, Apoptosis, Chemosensitizer

## Abstract

**Purpose:**

d,l-methadone (MET), an analgesic drug used for pain treatment and opiate addiction, has achieved attention from oncologists and social media as possible chemoensitizing agent in cancer therapy, notably brain cancer (glioblastoma multiforme, GBM). MET has been reported to enhance doxorubicin-induced cytotoxicity in GBM cells via activation of the µ-opioid receptor (MOR). Here, we extended this work and quantified the toxic effect of MET in comparison to other opioids alone and in combination with doxorubicin and the clinically more relevant alkylating drug temozolomide (TMZ), using a set of GBM cell lines and primary GBM cells.

**Methods:**

MOR expression in GBM cells was investigated by immunofluorescence and immunoblotting. Resistance to drugs alone and in combination with anticancer drugs was assessed by MTT assays. Concentration effect curves were fitted by nonlinear regression analysis and IC_50_ values were calculated. Apoptosis and necrosis rates were determined by annexin V/propidium iodide (PI)-flow cytometry.

**Results:**

MET alone was cytotoxic in all GBM cell lines and primary GBM cells at high micromolar concentrations (IC_50_ ~ 60–130 µM), observed both in the metabolic MTT assay and by quantifying apoptosis and necrosis, while morphine and oxycodone were not cytotoxic in this concentration range. Naloxone was not able to block MET-induced cytotoxicity, indicating that cell death-inducing effects of MET are not MOR-dependent. We recorded doxorubicin and TMZ concentration- response curves in combination with fixed MET concentrations. MET enhanced doxorubicin-induced cytotoxicity in only one cell line, and in primary cells it was observed only in a particular MET concentration range. In all assays, MET was not effective in sensitizing cells to TMZ. In two cell lines, MET even decreased the cell's sensitivity to TMZ.

**Conclusion:**

MET was found to be cytotoxic in GBM cells in vitro only at high, clinically not relevant concentrations, where it was effective in inducing apoptosis and necrosis. Sensitizing effects were only observed in combination with doxorubicin, but not with TMZ, and are dependent on cell line and the applied drug concentration. Therefore, our findings do not support the use of MET in the treatment of GBM in combination with TMZ, as no sensitizing effect of MET was observed.

**Supplementary Information:**

The online version contains supplementary material available at 10.1007/s00432-020-03485-3.

## Background

Grade IV glioma (glioblastoma multiforme, GBM) is the most aggressive form of brain cancer with the highest incidence among adults (Louis et al. 2016; Siegel et al. 2020). Median survival is 14.6 months under therapy, the 5-year survival rate is only less than 6% (Ostrom et al. 2016), indicating that there is high need for new therapeutic options. Therapeutic standard of care in GBM treatment is radiotherapy with concomitant temozolomide (TMZ) treatment (Stupp et al. 2005, 2009). Previous studies indicate that d,l-methadone (MET), an analgesic drug used for pain treatment and opiate addiction (Krantz and Mehler 2004; Parsons et al. 2010), increases apoptosis of leukemia cells and the cytotoxic effects of the topoisomerase II-inhibitor doxorubicin (Friesen et al. 2008, 2013; Singh et al. 2011). In a follow-up study, the same group showed that the opioid has also the potential to enhance apoptosis induced by doxorubicin in GBM cells (Friesen et al. 2014). The proposed mechanism of action involves activation of the µ-opioid receptor (MOR) and subsequent suppression of cAMP/protein kinase A (PKA) signaling via inhibitory G-proteins (Gi), which finally activates caspases and induces apoptosis. However, cAMP displays pro- and anti-apoptotic effects depending on cell type (Insel et al. 2012), raising the question if all GBM cells respond equally to MET treatment. In addition, it was shown that MET increases intracellular doxorubicin levels probably by inhibiting P-glycoproteins (P-gp) in GBM cells (Friesen et al. 2014). On the other hand, data published by others indicate no sensitizing effect of MET on various doxorubicin-treated canine tumor cells (Cueni et al. 2020).

It is important to note that the first-line therapy in GBM treatment is TMZ in combination with radiotherapy, while doxorubicin in first place has no indication for GBM because of its poor blood–brain barrier penetration and neurologic side effects (Merker et al. 1978; Neuwelt et al. 1981). A liposomal formulation of doxorubicin (Caelyx®) is sometimes used off-label with marginal benefit (Fabel et al. 2001; Fiorillo et al. 2004). Despite the lack of preclinical and clinical data on any beneficial effects of MET on TMZ therapy, it has been promoted as promising therapeutic option for GBM treatment.

Considering the importance of TMZ in GBM therapy, we aimed at investigating, first, the cytotoxic effect of MET in a variety of GBM cell lines differing in their p53 status and primary GBM cells and, secondly, to assess the contribution of MET to doxorubicin and TMZ-induced cytotoxicity in these cells. We recorded concentration–response curves in 3-(4,5-dimethylthiazol-2-yl)-2,5-diphenyltetrazolium bromide (MTT) assays of MET alone and doxorubicin or TMZ in combination with MET to quantify effects and determine IC_50_ values. Furthermore, we compared MET with morphine and oxycodone, other opioids used in oncology, and assessed the effect of the MOR inhibitor naloxone to determine if the opioid-induced cytotoxicity is mediated via MOR. Although MET was cytotoxic at high, clinically not relevant concentrations in all GBM cells, we only observed a weak MET-induced sensitization to doxorubicin in one established cell line and in primary cells, while no effect of MET was observed on TMZ-induced cell death.

## Methods

### Materials

All compounds were purchased from Sigma-Aldrich (St. Louis, MO, USA). Temozolomide (Temodal®) was dissolved in dimethyl sulfoxide (DMSO), doxorubicin hydrochloride, d,l-methadone hydrochloride, morphine sulfate salt pentahydrate, oxycodone, naloxone hydrochloride dehydrate were dissolved in sterile water.

### Cell culture

U87-MG, U251-MG, and U373-MG (Uppsala) GBM cell lines were obtained by Sigma-Aldrich (St. Louis, MO, USA) (HPA Culture Collections). The A172 GBM cell line was obtained by American Type Culture Collection (ATCC, Manassas, VA, USA). Cell lines were not used beyond passage 20. U251 Cells were cultivated in Roswell Park Memorial Institute (RPMI) 1640 medium (Biochrom, Berlin, Germany) containing 10% fetal calf serum (FCS; Biochrom, Berlin, Germany), 100 IU/ml penicillin, and 100 µg/ml streptomycin (Biowest, Nuaillé, France). All other cells and cell lines were cultivated in Dulbecco’s Modified Eagle Medium (DMEM) low glucose (Biowest, Nuaillé, France) containing 10% FCS (Biochrom, Berlin, Germany), 100 IU/ml penicillin, and 100 µg/ml streptomycin (Biowest, Nuaillé, France). Primary GBM cells derived from a primary GBM tumor biopsy were obtained from the University Hospital Cologne, genetically characterized and cultured as previously described (Haas et al. 2018).

### Western blot analysis

In order to prepare whole cell lysates, cells were washed with ice-cold phosphate-buffered saline (PBS; Biowest, Nuaillé, France) and lysed with ice-cold Denaturing Cell Extraction Buffer (FNN00091, Thermo Fisher Scientific, Waltham, MA, USA), incubated on ice for 30 min, and centrifuged for 15 min at 4 °C. The supernatant was used for protein content determination and subsequent immunoblotting. For immunoblotting standard procedures using the following antibodies were used as previously described (Haas et al. 2009). Anti-MOR-1 (D-12; 1:1000, Santa Cruz Biotechnology, TX, USA) combined with goat antimouse IgG-HRP (1:5000, Santa Cruz Biotechnology, TX, USA) and β-Actin-HRP (C-4; Santa Cruz Biotechnology, TX, USA). Immunoblots were developed with the enhanced chemoluminescence system (Amersham Biosciences, Little Chalfont, United Kingdom).

### MTT assay

For MTT assays, we followed a published protocol (Eckstein et al. 2009). Briefly, 5000 (A172, U251, U87, U373) or 15,000 (primary) cells were plated on 96 wells and grown at 37 °C and 5% CO_2_ overnight. Cell survival after exposure to either opioids alone or doxorubicin/TMZ in the presence of MET as indicated was determined by MTT assays after 72 h. In combination experiments MET or naloxone were added to culture medium 1 h prior to addition of compounds for 72 h. Controls were treated with vehicle (DMSO or water). Final DMSO concentrations in media did not exceed 1%.

### Annexin V/propidium iodide (PI) apoptosis assay

For apoptosis measurements the BD Pharmingen™ FITC Annexin V Apoptosis Detection Kit (BD Biosciences, Franklin Lakes, NJ, USA) was used according to the manufacturer’s protocol. Briefly, 2.5 × 10^5^ cells were seeded into 6-well plates and incubated at 37 °C and 5% CO_2_ overnight. After compound treatment for 72 h, cells were trypsinized and centrifuged for 4 min at 1500 × *g*. Supernatant was removed, and cells were resuspended in 500 µL binding buffer. 5 µL PI and 5 µL Annexin V-FITC were mixed with 100 µL of cells in binding buffer. After 15 min of incubation on ice, samples were analyzed by flow cytometry (FACSCaliburTM, BD Bioscience, Franklin Lakes, NJ, USA).

### Fluorescence microscopy

75,000 cells/mL were seeded on cover glasses and incubated at 37° C and 5% CO_2_ for 48 h before staining. Thereafter, cells were washed twice for 1 min with PBS and fixed with 4% paraformaldehyde/PBS for 10 min. Fixed cells were washed for 3 times with PBS and blocked in 2% bovine serum albumin (BSA; Sigma-Aldrich, St. Louis, MO, USA) including 0.2% Triton X-100 for 30 min. Thereafter, cells were incubated with MOR-1-antibody (D12, Santa Cruz Biotechnology, TX, USA) in 2% BSA (1:50) at 4° C overnight. The next day cells were washed incubated with antimouse IgG (H + L) DyLight™ 680 Conjugate (Cell Signalling Technology, Danvers, MA, USA) in 2% BSA (1:500) for 1 h. Finally, stained cells were washed for 3 times with PBS and covered with a coverslip using 1 drop of mounting medium. Cells stained with secondary antibody only were used as negative control. MOR expression was visualized using a Zeiss LSM 780 microscope (Carl Zeiss AG, Oberkochen, Germany) and 40 × magnification.

### Data analysis and statistical methods

Concentration effect curves were fitted to data points by nonlinear regression analysis using the four-parameter logistic equation (GraphPad™ Prism). Top of each curve was defined as 100% and bottom as 0%. Statistical differences between two groups were determined by paired 2-tailed Student’s *t* test. Comparisons among several groups were performed by ANOVA followed by Tukey post-hoc test. The data are presented as mean ± SEM or ± SD as indicated.

## Results

**Fig. 1:**
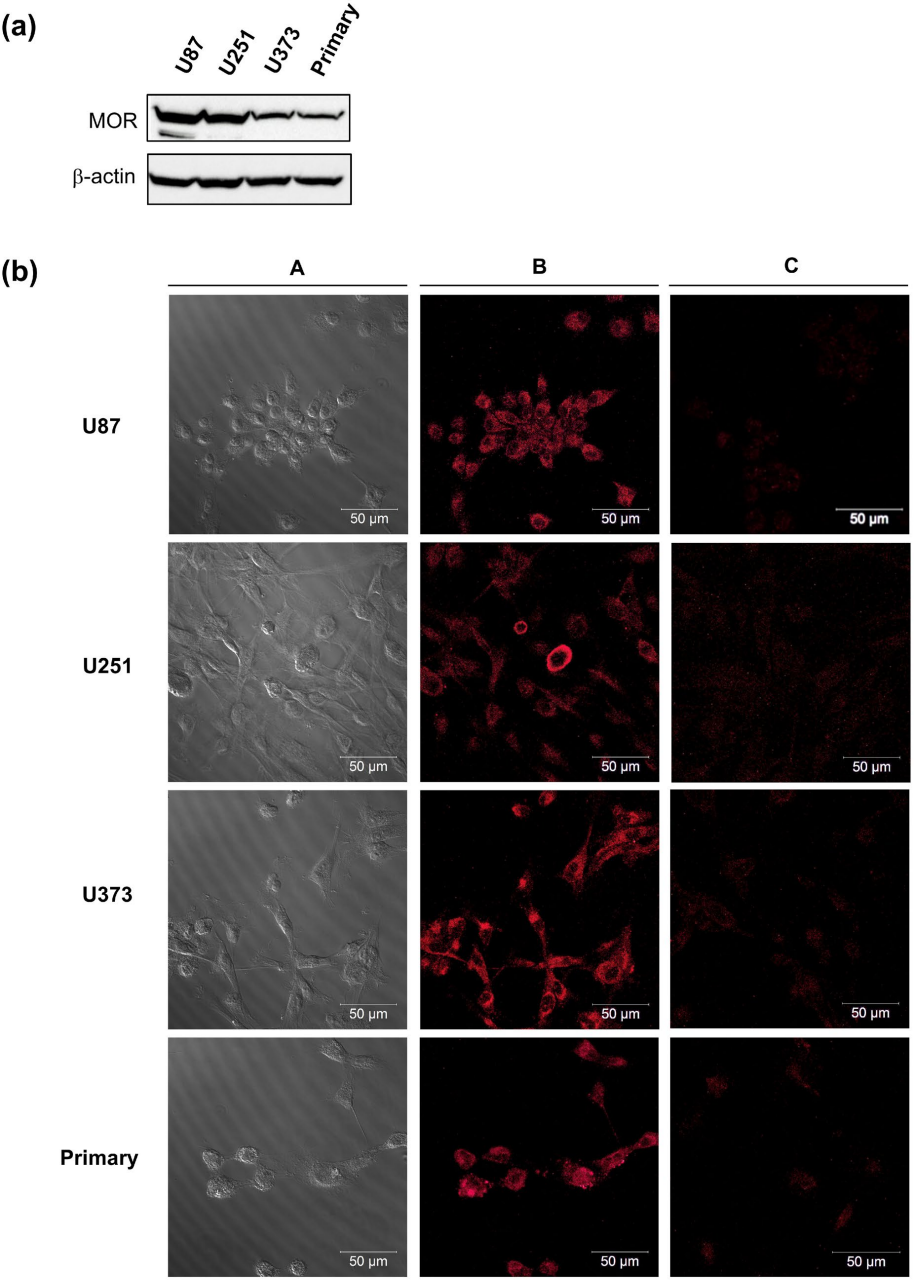
µ-Opioid receptor (MOR) expression in glioblastoma cells. **a** Representative Western blot showing protein expression of MOR in U87, U251, U373 GBM cell lines, and primary cells. β-actin blots were performed for loading control. **b** Representative immune fluorescence images showing expression of MOR in U87, U251, U373 GBM cell lines, and primary GBM cells. A, bright field images of respective cell lines; B, staining with primary and secondary antibody; C, treatment with secondary antibody only (negative control)

It has previously been shown that MOR is expressed in human GBM cell lines and primary cells (Brawanski et al. 2018; Friesen et al. 2014; Oppermann et al. 2019; Vatter et al. 2020). Here, we demonstrate the expression of MOR in commonly used GBM cell lines (U87, U251, U373) and primary GBM cells isolated from a patient biopsy by Western blotting (Fig. 1a) and immunocytochemistry (Fig. 1b), confirming and extending the finding that MOR is expressed in a wide range of GBM cells to a similar extent.

**Fig. 2:**
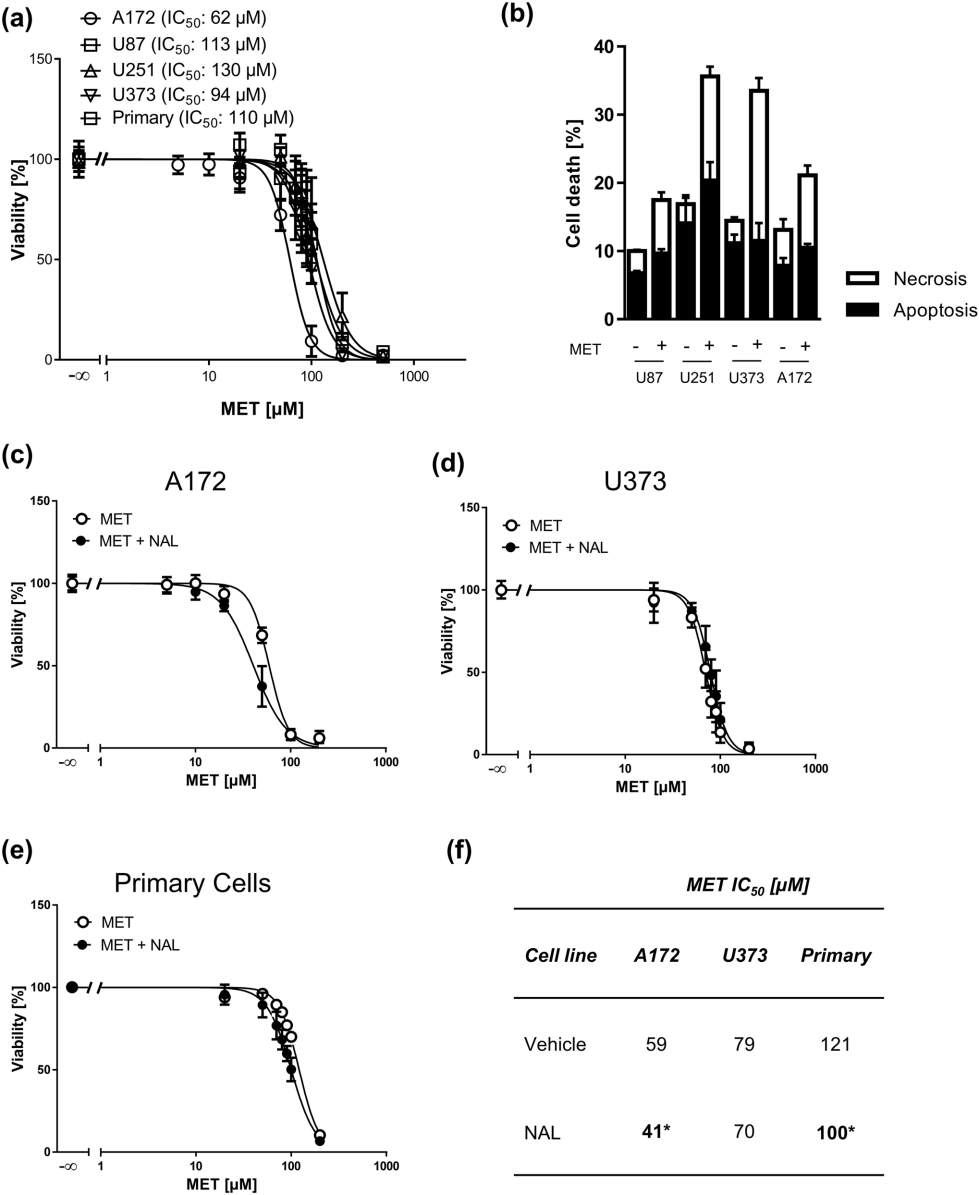
Cytotoxicity of methadone (MET) in GBM cells. **a** 3-(4,5-Dimethylthiazol-2-yl)-2,5-diphenyltetrazolium bromide (MTT) assays of four GBM cell lines and primary cells treated with increasing concentrations of MET for 72 h as indicated. Values are displayed as mean ± SD (*n* = 3). **b** AnnexinV/propidium iodide (AV/PI) FACS analysis of GBM cell lines U87, U251, U373, and A172 treated with an IC_50_ MET concentration for 72 h. AV positive cells were considered apoptotic and PI positive/AV negative cells necrotic. Values are displayed as mean ± SEM (*n* = 4). MTT assays of A172 (**c**), U373 (**d**) GBM cell lines and primary cells (**e**) treated with increasing concentrations of MET for 72 h pretreated with 300 µM naloxone (NAL) for 1 h. Values are displayed as mean ± SD (*n* = 3). **f** IC_50_ values of MET in GBM cells derived from MTT assays; *p < 0.05

We next asked whether MET is cytotoxic in these MOR-expressing cells, including the A172 cell line in which beneficial effects of MET on doxorubicin-induced cytotoxicity were reported (Friesen et al. 2014). In MTT viability assays, we obtained very similar IC_50_ values for MET in all GBM cell lines, which were in the range of 62–130 µM, measured 72 h after the onset of treatment. MET displayed a steep concentration response in all cells (Hill slopes ranging from − 3.3 to − 4.4). The lowest IC_50_ of 62 µM was obtained for A172 cells (Fig. 2a). Despite differences in p53 status (Table S1), all cell lines were uniformly responding to MET. Annexin V/propidium iodide (AV/PI) flow cytometry analysis of cell lines treated with the previously determined IC_50_ MET concentrations revealed that approximately 50% of cell deaths each account for apoptosis and necrosis (Fig. 2b).

In order to test if MET-induced cytotoxicity is mediated via MOR, we co-treated A172, U373 and primary cells with the MOR antagonist naloxone and MET. Naloxone treatment alone did not affect cell viability and, most importantly, it was not capable of abolishing MET-induced cytotoxicity (Fig. 2c–e). The opposite was true, naloxone even significantly increased MET toxicity in A172 and primary cells (Fig. 2f), indicating that MET does not require MOR for its cytotoxic action.

**Fig. 3:**
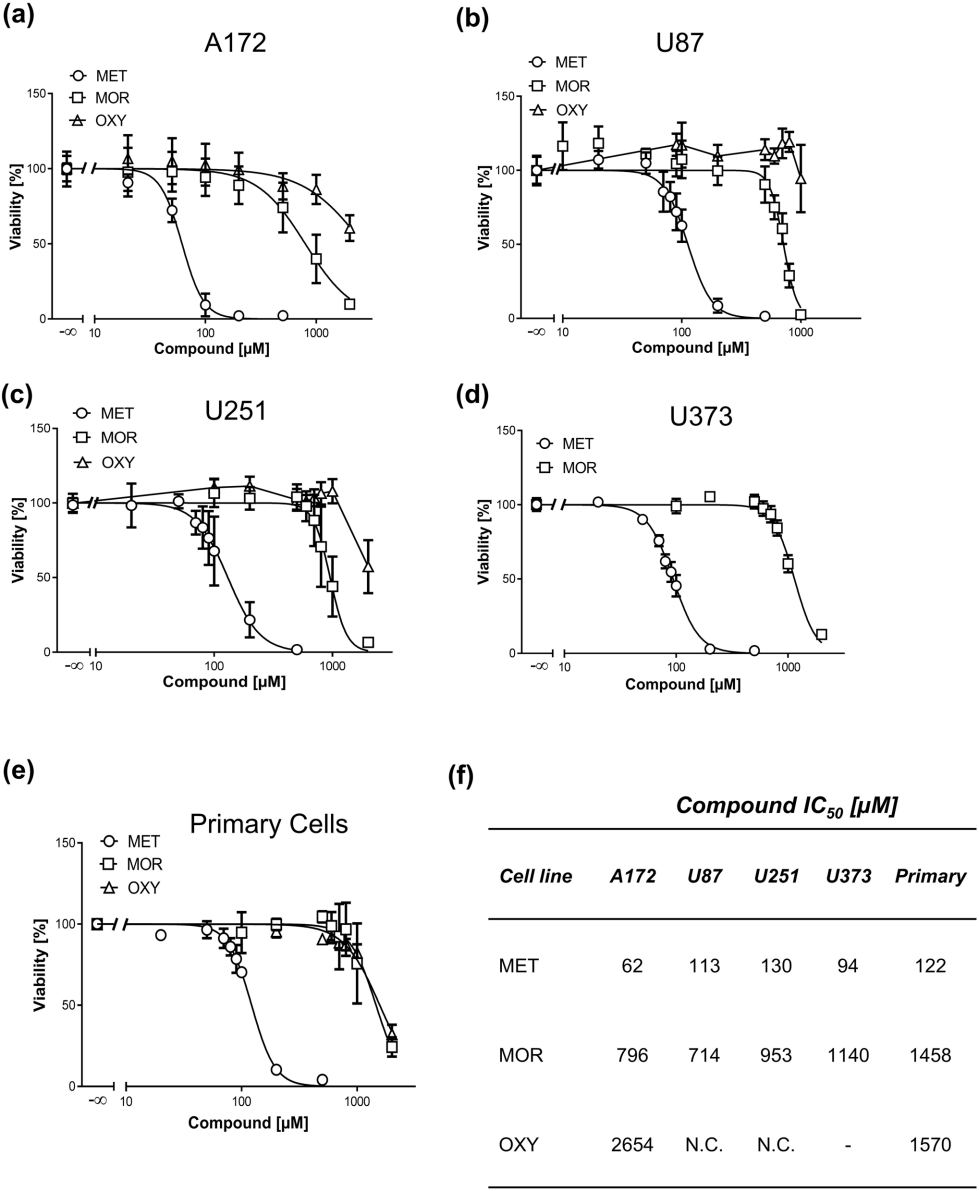
Comparison of opioid-induced cytotoxicity in GBM cell lines and primary cells. MTT assays of A172 (**a**), U87 (**b**), U251 (**c**), U373 (**d**), GBM cell lines and primary cells (**e**) treated with increasing concentrations of MET, morphine (MOR), and oxycodone (OXY) for 72 h as indicated. Values are displayed as mean ± SD (*n* = 3–4). **f** IC_50_ values of MET, MOR, and OXY in GBM cells derived from MTT assays, *NC* not calculated, – not determined

To further verify these findings, we treated cells with other MOR agonists used in clinical practice for pain management such as morphine and oxycodone. Strikingly, both compounds showed very weak cytotoxicity in supra-therapeutic concentrations in all GBM cell lines and primary cells (Fig. 3a–e). The determined IC_50_ values were in the millimolar range or could not be established because of very low cytotoxicity (Fig. 3f).

**Fig. 4:**
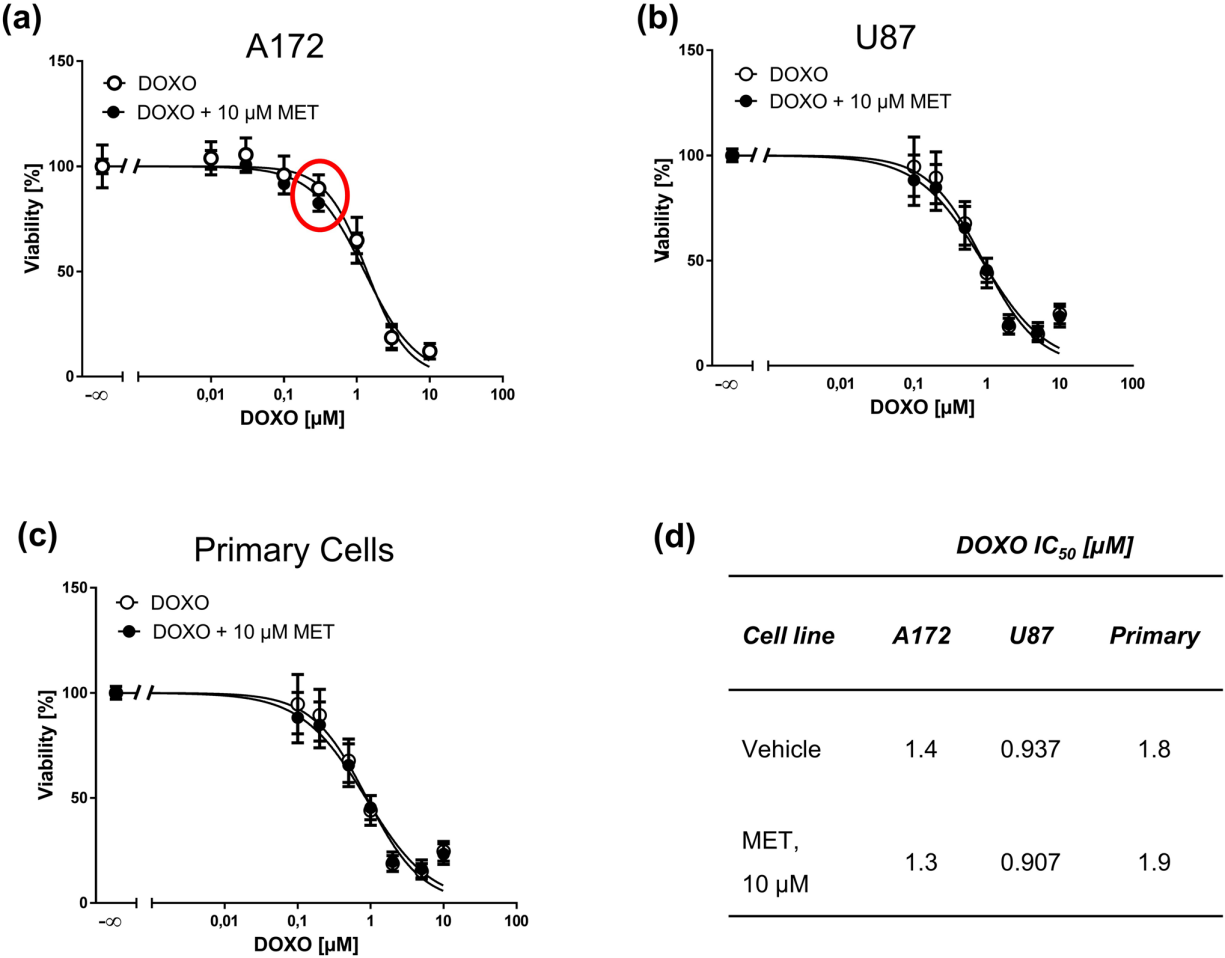
MET (10 µM) does not sensitize GBM cell lines and primary cells to doxorubicin (DOXO). MTT assays of A172 (**a**), U87 (**b**) GBM cell lines and primary cells (**c**) treated with increasing concentrations of DOXO for 72 h pretreated with vehicle or 10 µM MET for 1 h. **d** IC_50_ values derived from MTT assays of doxorubicin treated GBM cells with or without 10 µM MET. Values are displayed as mean ± SD (*n* = 3). Note: in A172 at one data point of combination treatment differed from DOXO treatment [0.3 µM, red circle in (**a**)]

Previously, it was reported that various MET concentrations (e.g. 10, 3, 1 µg/mL corresponding to ~ 30, 10, 3 µM, respectively) were capable of sensitizing A172 cells to a fixed doxorubicin concentration (0.3 µg/mL = 0.5 µM) in apoptosis assays (Friesen et al. 2014). In order to determine a concentration relationship, we treated A172, U87, and primary cells with ascending doxorubicin concentrations combined with 10 µM MET, a concentration which was not cytotoxic, but close to what might be reached in plasma of patients. We chose U87 cells because they do not express P-gp (Haas et al. 2018), while A172 cells likely express P-gp as its inhibtion by MET was reported to be responsible for doxorubicin accumulation (Friesen et al. 2014). MTT concentration–response curves overlapped in both tested GBM cell lines and, interestingly also in primary GBM cells (Fig. 4a–c); respective IC_50_ values showing no significant differences are displayed in Fig. 4d. Of note, in A172 cells at 0.3 µM doxorubicin, combination treatment with 10 µM MET slighly reduced cell viability as compared to doxorubicin treated cells alone (red circle in Fig. 4a). This is close to the concentrations tested in a previous work (0.5 µM doxorubicin combined with 3–30 µM MET), where effects on apoptosis were observed (Friesen et al. 2014).

**Fig. 5:**
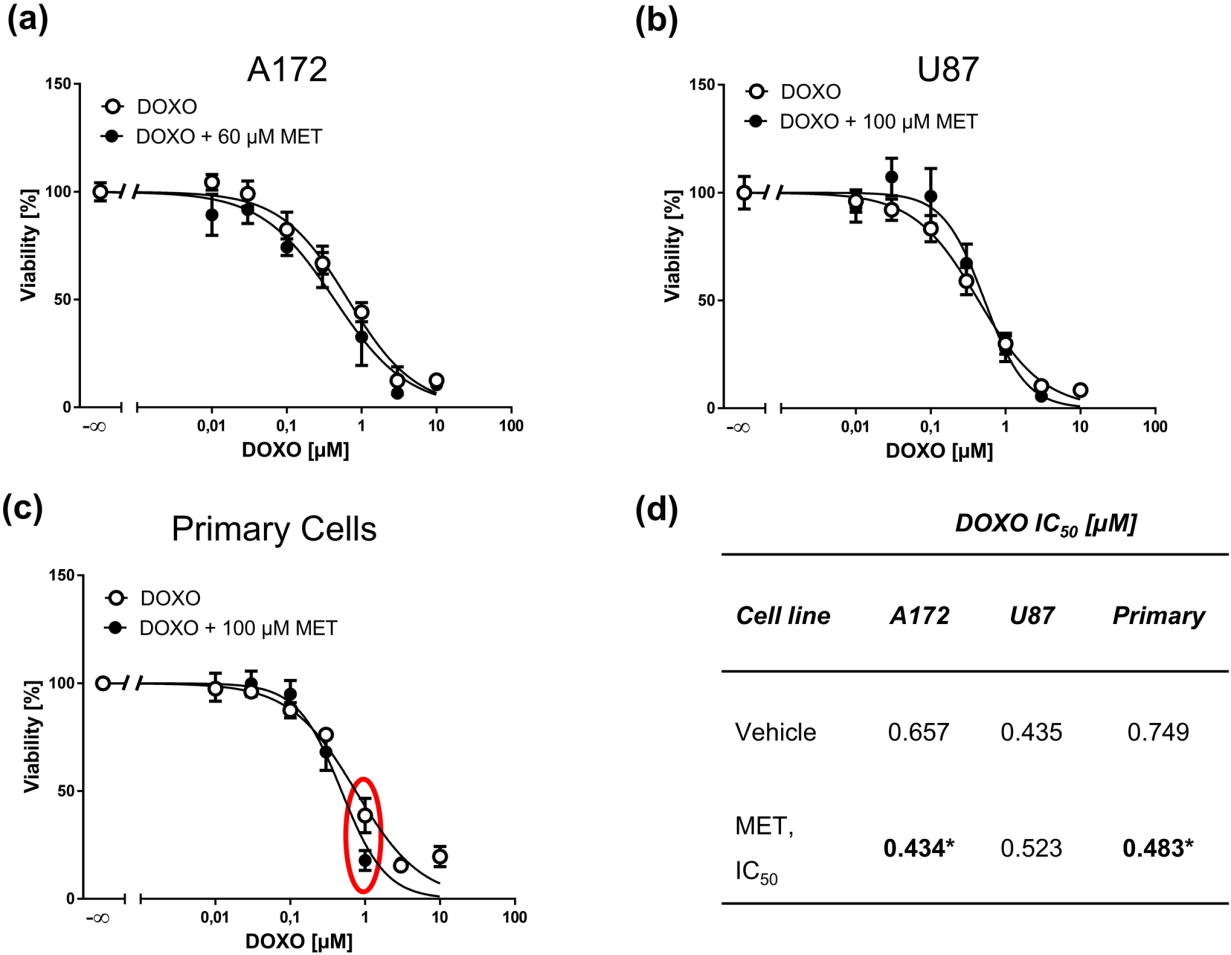
Sensitization of GBM cells by high MET concentrations to DOXO is cell line-dependent. MTT assays of A172 (**a**), U87 (**b**) GBM cell lines, and primary cells (**c**), treated with increasing concentrations of DOXO for 72 h pretreated with vehicle or the corresponding IC_50_ concentration of MET for 1 h. Values are displayed as mean ± SD (*n* = 3) (**d**) IC_50_ values derived from MTT assays of DOXO-treated GBM cells with or without an IC_50_ concentration of MET; *p < 0.05. Note: Combination treatment in primary GBM cells was only statistically different from DOXO alone treated cells due to one data point [red circle in (**c**)]

Next, we repeated this experiment with previously determined IC_50_ concentrations of MET (Fig. 2a). High dose MET treatment (60 µM) resulted in a slight left shift of the doxorubicin concentration–response curve in A172 cells (Fig. 5a), while curves overlapped in U87 cells at 100 µM MET (Fig. 5b). Also in primary GBM cells at 100 µM MET curves largely overlapped independently of treatment (Fig. 5c). Statistical analysis revealed that MET treatment significantly reduced doxorubicin IC_50_ values in A172 and primary GBM cells indicating a sensitization (Fig. 5d). Looking closer at the curves of primary GBM cells, the data revealed at both curves overlap at most data points except at 1 µM (~ 0.5 µg/mL) doxorubicin (red circle in Fig. 5c), which explains the difference in IC_50_ values and indicates that a potential synergism of MET and doxorubicin highly depends on cell line and applied drug concentrations.

**Fig. 6:**
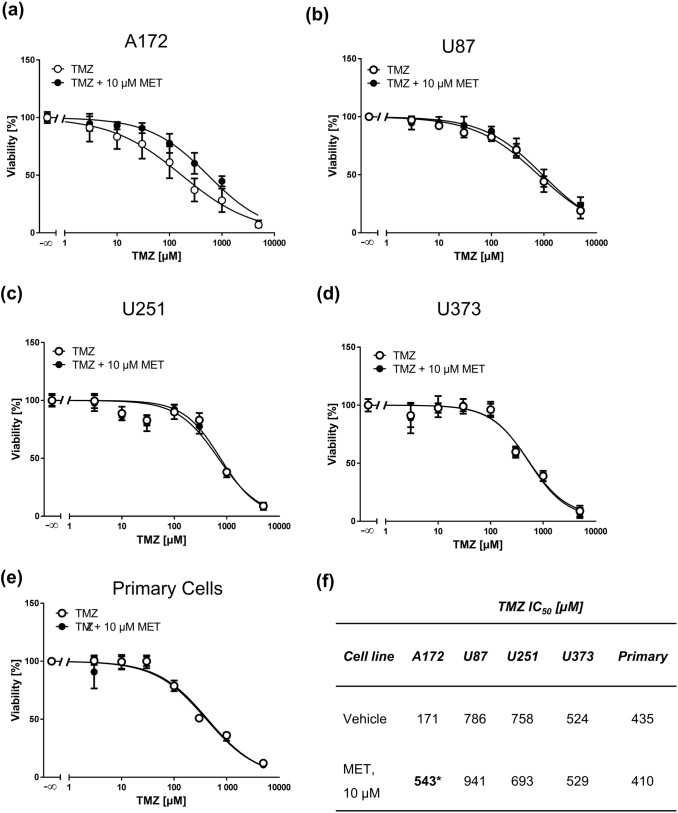
MET (10 µM) does not sensitize GBM cell lines and primary cells to TMZ. MTT assays of A172 (**a**), U87 (**b**), U251 (**c**), U373 (**d**) GBM cell lines and primary cells (**e**) treated with increasing concentrations of TMZ for 72 h pretreated with vehicle or 10 µM MET for 1 h. Values are displayed as mean ± SD (*n* = 3–4). (**f**) IC_50_ values of TMZ (vehicle) and TMZ + MET-treated GBM cells derived from MTT assays; *p < 0.05

**Fig. 7:**
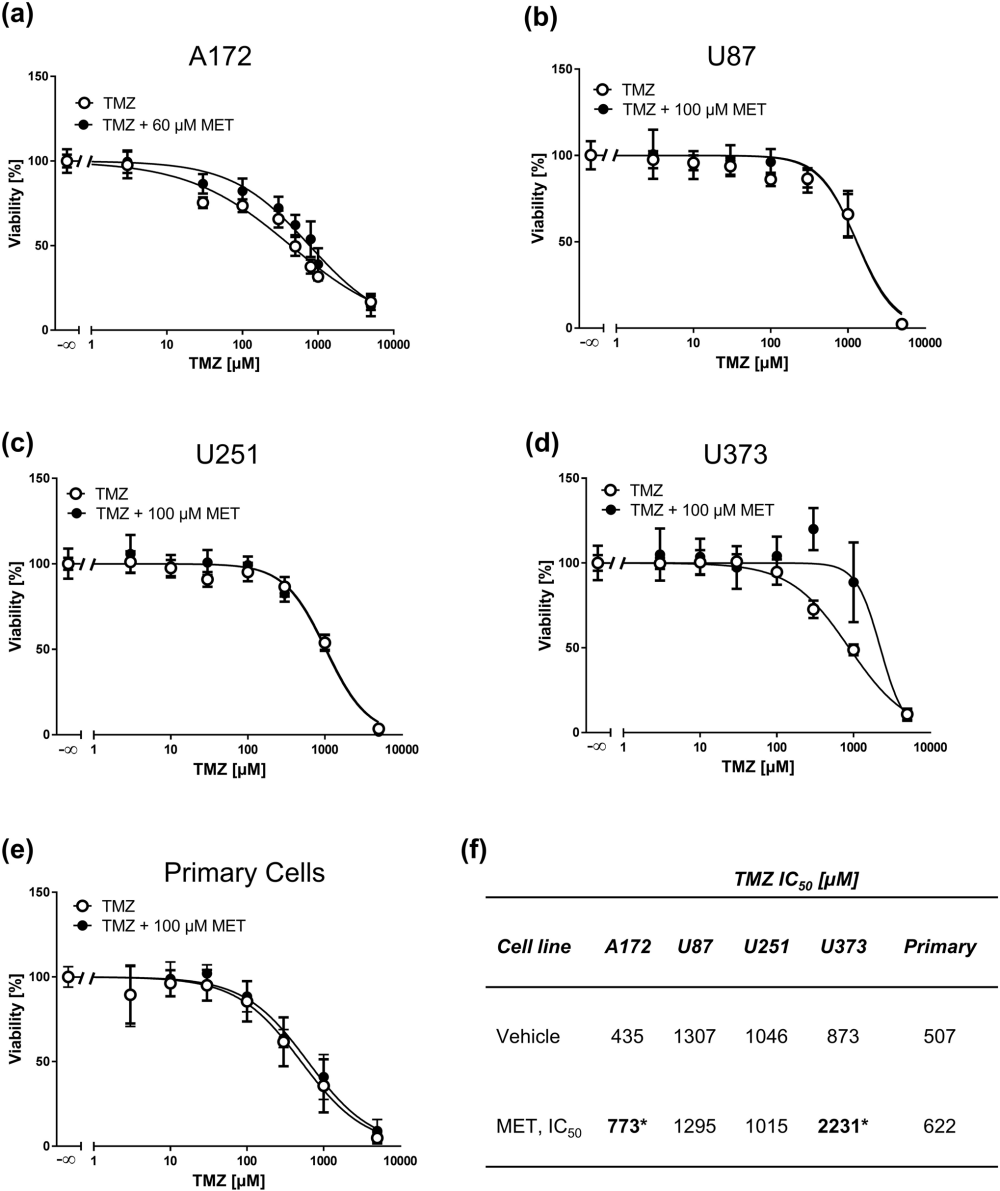
High MET concentrations do not sensitize GBM cell lines and primary cells to TMZ. MTT assays of A172 (**a**), U87 (**b**), U251 (**c**), U373 (**d**) GBM cell lines and primary cells (**e**) treated with increasing concentrations of TMZ pretreated with vehicle or the corresponding IC_50_ concentration of MET for 1 h. Values are displayed as mean ± SD (*n* = 3–4). **b** IC_50_ values of TMZ (vehicle) and TMZ + MET-treated GBM cells derived from MTT assays; *p < 0.05

Doxorubicin is not used in the first-line treatment for GBM. Therefore, we studied the effect of MET on the toxicity of TMZ, which is the clinically relevant drug used in GBM therapy. We treated four GBM cell lines and primary GBM cells with ascending TMZ concentrations in combination with 10 µM MET and performed MTT assays. In A172 cells MET even reduced the sensitivity to TMZ (Fig. 6a) while in all other cells no effect was observed (Fig. 6b–e). The only statistical difference in IC_50_ values was obtained for A172 cells (Fig. 6f) confirming the negative impact of MET on TMZ treatment in this cell line. Similar results were obtained when cells were treated with an IC_50_ MET concentration in combination with TMZ (Fig. 7). At this high MET concentrations, both A172 and U373 (Fig. 7a, d, respectively) cells responded in a less sensitive way to TMZ.

## Discussion

MET is a widely used therapeutic opioid in narcotic addiction and neuropathic pain syndromes. In oncological settings, it is regularly used as a long-lasting analgesic. Recently, it has also been proposed as a chemosensitzing agent in leukemia and GBM therapy based on the results of in vitro studies and a xenograft mouse model (Friesen et al. 2014, 2008, 2013). In these studies, fixed doxorubicin concentrations combined with MET were applied and MET was found to be capable of sensitizing cells to doxorubicin in terms of apoptosis induction. The proposed mechanism of action rests on activation of MOR, inhibition of P-gp by MET leading to increased intracellular doxorubicin levels, and subsequent induction of apoptosis (Friesen et al. 2014). In another study, the data were not reproduced in doxorubicin and MET-treated canine tumor cells (Cueni et al. 2020), indicating a need for further in vitro studies. MET has also been implicated in enhancing cytotoxic effects of other chemotherapeutics in cancer cell lines of different origins, such as bladder cancer, squamous cell carcinoma and head and neck cancer, albeit with varying efficacy, greatly depending on cell type, the chemotherapeutic agent and applied concentrations (Landgraf et al. 2019; Michalska et al. 2018; Shi et al. 2019). Also for GBM, conflicting results were reported. Thus, a sensitization to acid‐based photodynamic therapy (ALA‐PDT) by MET was observed in the GBM cell line A172 (Shi et al. 2019, 2020), indicating that MET might also be effective in GBM in combination with other treatments apart from doxorubicin. When MET was combined with the more relevant treatment TMZ and irradiation (up to 8 Gy), no beneficial effect was observed in vitro (Oppermann et al. 2019; Vatter et al. 2020), while another study reported agonistic and antagonistic effects at high MET concentrations (> 15 µg/mL) on TMZ, which was cell line-dependent (Brawanski et al. 2018). Interestingly, in these studies, MET effects on GBM cells were independent of MOR expression levels raising the question if MOR alone mediates the cytotoxic action of MET.

In our work presented here, we wished to quantify first whether MET alone is cytotoxic in GBM cells. MET induced cell death in a similar concentration range in various cell lines and primary cells. We also found that at high dose levels of MET (IC_50_ concentrations) apoptosis and necrosis were induced to a similar extend in most cell lines, except for U373 cells where about two thirds of cell deaths accounted for necrosis. This is in line with what has been shown by others, i.e., MET is able to induce apoptosis (Friesen et al. 2014) or necrotic-like cell death in cells of neuronal origin (Perez-Alvarez et al. 2010, 2011). Interestingly, we observed that naloxone was not able to block MET-induced cytotoxicity and other MOR agonists, morphine and oxycodone, were hardly cytotoxic at clinically relevant concentrations. This implies that MET's cytotoxic action on GBM cells is not MOR-dependent. MET might interfere with other cellular targets, which are responsible for its cytotoxicity. For instance, MET, in contrast to oxycodone and morphine, inhibits at low micromolar concentrations members of the voltage-gated potassium channel family (Fanoe et al. 2009; Katchman et al. 2002; Zunkler and Wos-Maganga 2010). Pharmacological inhibition of these channels leads to cell death in various tumor cells, including GBM cell lines (Sales et al. 2016) and thus the channels were proposed to be promising targets for cancer treatment (Wang et al. 2017). Whether MET-induced inhibition of voltage-gated potassium channels at the concentrations used in our work leads to cytotoxicity in GBM cells needs further investigation.

As conflicting results have been reported on the capability of MET to sensitize tumor cells to doxorubicin, we tested the combination of doxorubicin and MET in our cellular system. We used an approach commonly applied to determine sensitizing effects of a compound on cytostatic drugs. We treated cells with a wide range of doxorubicin combined with low (10 µM) and high concentrations (determined IC_50_ concentration for each cell line) of MET for 72 h because at this time point MET already induced cell death. We found a sensitizing effect only for the A172 cell line and to some extent in primary GBM cells, but not for the U87 cell line. We have previously shown that P-gp is not expressed in U87 cells (Haas et al. 2018), which might explain that these cells cannot be sensitized to doxorubicin by MET. Whether inhibition of doxorubicin efflux, as reported for the A172 cell line (Friesen et al. 2014), or inhibition of voltage-gated potassium channels is the underlying reason of MET-induced apoptosis/necrosis remains an open question.

Another important finding of our study is that the sensitizing effect of MET in A172 and primary cells was highly dependent on the applied drug concentrations. Although a complete left shift of the doxorubicin concentration–response curve upon MET treatment only was observed at high, clinically not relevant MET concentrations (60 µM) in A172 cells, lower MET concentrations (10 µM) only further increased doxorubicin cytotoxicity at one single doxorubicin/MET concentration combination but not over the whole concentration range. This might have clinical implications as it is challenging to exactly achieve the needed doxorubicin and MET levels in vivo where synergistic cell death-inducing effects might occur. In addition, tolerable MET plasma levels in addicts are between 0.3 and 1.3 µg/ml (corresponding to ~ 1–4 µM) after a dose of 60–120 mg/day (Dole and Kreek 1973; Inturrisi et al. 1987) which is much lower as compared to effective in vitro MET concentrations reported previously (Friesen et al. 2014) and in our present study. When a blood–brain barrier penetration of only 42% for MET is assumed (Oldendorf et al. 1972), sufficient brain levels can hardly be reached in GBM patients at feasible MET doses. It could be argued that MET accumulates in tissue stores and also in brain and corresponding tumors (Linares et al. 2015). However, if sufficient MET levels are actually reached in brain tumor tissue at clinically achievable doses warrants further investigation, apart from the fact that doxorubicin is not the indicated treatment option for GBM due to its low blood–brain barrier penetration and neurological side effects (Merker et al. 1978; Neuwelt et al. 1981). Although pegylated and liposomal-encapsulated formulations of doxorubicin (Caelyx®) with increased brain uptake and less side effects are available, GBM is rarely treated with doxorubicin and its liposomal formulations in clinical practice (Fabel et al. 2001; Fiorillo et al. 2004).

Because of these shortcomings, we also tested the clinically more relevant drug TMZ in combination with MET. We did not observe an effect of MET on TMZ-induced cytotoxicity in none of the tested GBM cell lines and primary cells, which is in line with a previous study (Oppermann et al. 2019). Importantly, in U373 and A172 cells MET even reduced sensitivity to TMZ, an effect that has also been demonstrated by others in different GBM cell lines (Brawanski et al. 2018). This might be due to cell cycle inhibition following MET treatment especially at high cytotoxic concentrations, which counteracts TMZ-induced cell death responses (Roos et al. 2004). In contrast to other studies where MET and TMZ were applied for 6 days (Brawanski et al. 2018; Kaina et al. 2020; Oppermann et al. 2019), we applied a 72-h MTT protocol as at this time MET already displayed high cytotoxicity and, therefore, we considered it sufficient to observe an effect on TMZ. As a consequence, the determined TMZ IC_50_ values in our study are in the higher micromolar range, as TMZ requires repeated cell cycles to process lesions and activate cell death pathways (He and Kaina 2019). Despite these differences in treatment protocols we yielded similar results, which strengthen the conclusion also drawn by other groups that MET is not capable to synergistically sensitize GBM cell to TMZ. Similar data were obtained on glioblastoma cells and TMZ-induced apoptosis and cellular senescence, demonstrating that MET does not impact pathways involved in these endpoints (Kaina et al. 2020).

## Conclusions

We conclude that the cytotoxic effect of MET alone on GBM cells is not mediated via the opioid receptor MOR. This implicates that other cellular targets, including voltage-gated potassium channels, are involved, which warrants further investigation. Furthermore, our findings do not support the use of MET in the treatment of GBM in combination with TMZ, as no sensitizing effect of MET was observed. In GBM therapy, treatment with doxorubicin is not the rule. Although we observed in two cell lines (out of 3) a supportive effect of MET on doxorubicin-induced cytotoxicity, we doubt that critical MET concentration levels might be reached in the brain of patients to achieve a potential synergistic effect with doxorubicin. It is obvious that further clinical studies are warranted.

## Supplementary Information

Below is the link to the electronic supplementary material.

Supplementary file1 (DOCX 14 KB)

## Data Availability

The datasets used and/or analyzed during the current study are available from the corresponding author on reasonable request.

## Abbreviations

DOXO: Doxorubicin
GBM: Glioblastoma multiforme
Gi: Inhibitory G-protein
MET: D,l-methadone
MOR: µ-Opioid receptor
MTT: 3-(4,5-dimethylthiazol-2-yl)-2,5-diphenyltetrazolium bromide
NAL: Naloxone
OXY: Oxycodone
P-gp: P-Glycoprotein
PKA: cAMP-dependent protein kinase
PI: propidium idodide
TMZ: Temozolomide
