# Supplementary material for: Methadone-mediated sensitization of glioblastoma cells is drug and cell line dependent
Source: J Cancer Res Clin Oncol. 2020 Dec 14;147(3):779–92. doi: 10.1007/s00432-020-03485-3 (PMC7872955; doi:10.1007/s00432-020-03485-3)
Supplement: Supplementary file 1 — Supplementary file1 (DOCX 14 KB) [file 432_2020_3485_MOESM1_ESM.docx]

**Supplementary Table 1 MGMT and TP53 status of GBM cell lines**

| **Cell Line** | **MGMT Status (Promotor Methylation)** | **TP53 Status** |
| --- | --- | --- |
| A172 | Negative (methylated) ^1^ | wt ^2,3^ |
| U87-MG | Negative (methylated) ^1^ | wt ^2,3^ |
| U251-MG | Negative (methylated) ^1^ | Homozygous Missense Mutation (Arg -> His) ^2^ |
| U373-MG | Negative (methylated) ^1^ | Homozygous Missense Mutation (Arg -> His) ^2^ |

MGMT: methylguanine-DNA methyltransferase, TP53: tumour suppressor p53, wt: wild type

1. Hermisson, M.; Klumpp, A.; Wick, W.; Wischhusen, J.; Nagel, G.; Roos, W.; Kaina, B.; Weller, M. O6‐ methylguanine DNA methyltransferase and p53 status predict temozolomide sensitivity in human malignant glioma cells. J. Neurochem. 2006, 96, 766–776, doi:10.1111/j.1471‐4159.2005.03583.x.

2. Ishii, N.; Maier, D.; Merlo, A.; Tada, M.; Sawamura, Y.; Diserens, A.C.; van Meir, E.G. Frequent coalterations of TP53, p16/CDKN2A, p14ARF, PTEN tumor suppressor genes in human glioma cell lines. Brain Pathol. 1999, 9, 469–479.

3. Michaud, K.; Solomon, D.A.; Oermann, E.; Kim, J.‐S.; Zhong, W.‐Z.; Prados, M.D.; Ozawa, T.; James, C.D.; Waldman, T. Pharmacologic inhibition of cyclin‐dependent kinases 4 and 6 arrests the growth of glioblastoma multiforme intracranial xenografts. Cancer Res. 2010, 70, 3228–3238.
